# Technical challenges of studying the impact of plasma components on the efficacy of lipid nanoparticles for vaccine and therapeutic applications

**DOI:** 10.1038/s41467-024-47724-4

**Published:** 2024-05-09

**Authors:** Jens B. Simonsen

**Affiliations:** Jbsimonsen Consult, Virum, Denmark

**Keywords:** Biological physics, Drug delivery

**arising from** K. Liu et al. *Nature Communications* 10.1038/s41467-023-39768-9 (2023)

Recently, Liu et al.^1^. suggested to use magnetic beads coated with PEG-antibodies to pull down and isolate clinically relevant pegylated lipid nanoparticles (LNPs) from plasma. In principle, this method solves one of the major hurdles in the study of interactions between lipid-based nanometer-sized delivery systems for therapeutic use and blood constituents, namely co-isolation of similarly sized endogenous nanoparticles (NPs) with surface lipids including lipoproteins and extracellular vesicles (EVs). The interaction between exogenous lipid-based NPs and plasma constituents is believed to dictate the fate and, thus, the therapeutic efficacy and toxicity of these products. For these reasons, much effort is put into improving the study of the biomolecule adsorption to NPs. Here, I question whether the pull-down method proposed by Liu et al. indeed avoids the co-isolation of lipoproteins and EVs. My main concern is the inherent propensity of the labile pegylated-lipid, DMPE-PEG, incorporated into their LNPs to dissociate and become embedded into the surface of endogenous NPs. This type of dynamic may potentially pose a great challenge to affinity-based isolation methods. With that in mind, I discuss some of the data and conclusions presented by Liu et al.

LNPs have garnered much attention, as they have unlocked the clinical potential of nucleic acid therapeutics such as the efficacious mRNA-based covid-19 vaccines from Pfizer/BioNTech and Moderna and the siRNA-based product Onpattro® for the treatment of hereditary transthyretin amyloidosis. Despite their clinical successes, our understanding of how and which biological components in complex biofluids, including blood, LNPs interact with, and the impact of such interactions on the therapeutic efficacy and toxicity of LNP-based therapeutic products are still sparse and almost entirely limited to interactions with apolipoprotein E (apoE). To fully exploit the potential of LNPs for the delivery of nucleic acid-based therapies across various indications, a better understanding of the mechanisms by which LNPs interact with the biochemical environment they are intended to function in is necessary.

The study of proteins adsorbed to NPs during circulation, also referred to as the protein corona, has been a hot topic for many years in the drug delivery field. However, protein corona studies are very challenging, particularly when studying lipid-based NPs such as liposomes and LNPs. This is partly due to: (i) the coexistence of compositional, mass density, and size-similar endogenous NPs in blood plasma, including high-density lipoproteins (HDL), low-density lipoproteins (LDL), very low-density lipoproteins (VLDL), chylomicrons, and EVs complicating selective purification of exogenous lipid-based NPs from plasma^2^; and (ii) inherent limitations in tools used for NP characterization. These limitations are put into context below by discussing some of the key findings presented by Liu et al.

**Fig. 1:**
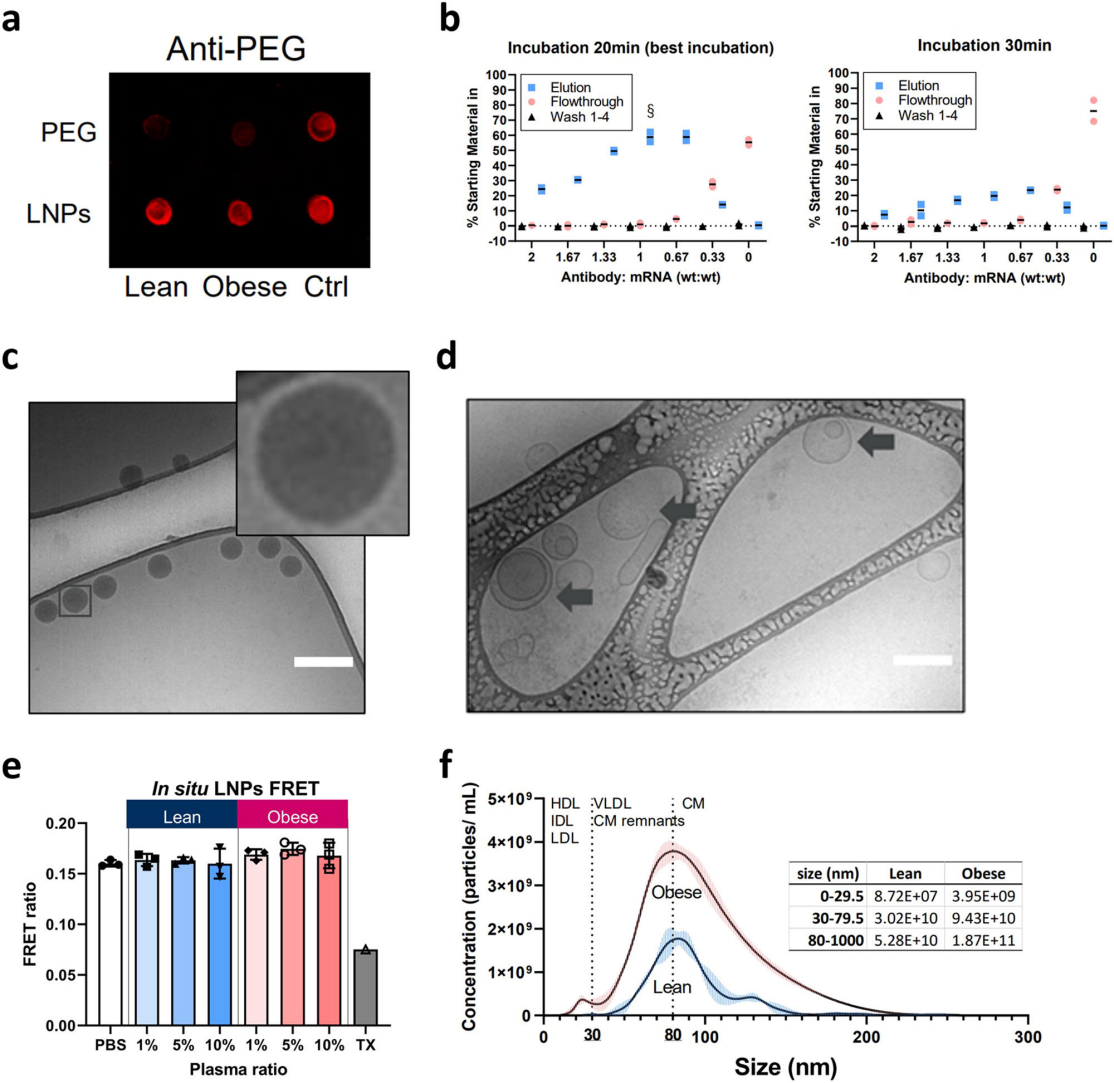
Key data from Liu et al.^1^*.* **a** Dot blots of PEG-specific affinity purification. “PEG-lipid or LNPs at an equivalent concentration to the 200 ng/well dose were spiked into lean and obese plasma.” (source: Supplementary Fig. 10e)^1^. **b** LNP recovery after incubating the 4 h pre-incubated LNPS with 1% OP with magnetic beads coated with PEG-antibodies for 20 and 30 min. (source: Supplementary Fig. 10b and c)^1^. **c** and **d** Cryo-TEM micrographs of the naked LNPs in buffer (source: Supplementary Fig. 3c)^1^ (c) and LNP isolated from the 1% OP sample (source: Supplementary Fig. 12b)^1^ (d). Scale bars = 200 nm. **e** FRET profiles were measured post 4 h incubation in 1–10% of lean pool and obese pool plasma. TX: 1% Triton X-100. Error bars represent standard deviation of the mean values derived from experimental replicates (*n* = 3) (source: Supplementary Fig*.* 8)^1^. **f** Size distribution of endogenous particles in LP and OP plasma determined by NTA, which largely overlap with LNP ( ~ 80 nm) (source: Supplementary Fig. 1)^1^.

To validate that their PEG affinity-based LNP isolation method avoids contamination by lipoproteins, EVs and other plasma components, Liu et al. analyzed pull-down fractions from rat blood plasma samples which were incubated with either pegylated LNPs or pure PEG-lipids (non-LNP control) with dot blots based on PEG-antibody staining (Fig. 1a)^1^. LNPs isolated from plasma from either an obese (OP) or lean (LP) rat model with the pull-down method exhibit strong PEG-antibody signals, while pull-down fractions from the non-LNP control only show faint signals. That said, the non-LNP(obese) control signal corresponds to 29% of the positive LNP(obese) anti-PEG signal according my simple ImageJ analysis of the dot blot images. Although the dot blot data may imply that PEG affinity-based LNP isolation avoids co-isolation of lipoproteins and EVs (the non-LNP control signals could potentially come from pull-down of single PEG-lipids or PEG-lipid micelles), it is important to stress that that the apparently faint signals in the non-LNP controls may also suggest that these fractions are not devoid of endogenous NPs from plasma. Hence, it is unclear whether the apparently faint signals from the controls refer to a limit or a significant amount of endogenous NPs with limited but sufficient PEG-lipid content to be captured.

The desorption of DMPE-PEG lipids from the LNPs that Liu et al. are studying is needed for the formation of a protein corona. The endogenous NPs may likely help facilitating the net DMPE-PEG desorption from LNPs into their lipid-based surfaces. On this note, exchange of dimyristoyl-based lipids between liposomes^3^ and net transfer of similarly lipid-tailored entities, dipalmitoyl-conjugated fluorophore lipids from one type of lipid-based NP to lipoproteins have been reported^4,5^. Importantly, several studies^6–8^ have shown that labile dimyristoyl-based PEG-lipids like DMPE-PEG promote rapid depegylation from LNPs in blood plasma, enable apoE adsorption onto depegylated LNPs. Taken together, the endogenous lipoproteins likely play a key role in serving as both PEG-lipid hosts and apoE sources. Liu et al. further demonstrated that the highest LNP yield could be achieved by incubating the capturing beads for 20 min. with LNPs that have been pre-incubated for 4 h with diluted plasma. Interestingly, longer bead incubation periods resulted in significantly decreased LNP-capturing (Fig. 1b)^1^. This observation could further support redistribution of DMPE-PEG from LNPs to plasma components when using a longer incubation time than 20 min with the capturing beads. Without any further contaminant-targeted characterization of the pull-down fractions, it remains uncertain to which extent down-stream characterization of protein content may be confounded by endogenous NPs.

The study by Liu et al. nicely highlights that the plasma composition has a huge impact on the proposed protein and lipid corona composition on LNPs as well as the potency of LNP (mRNA)-mediated eGFP expression in hepatocytes. Specifically, they study the impact of plasma from lean and obese rats on the LNP transfection potency and identify HDL-LNP interactions as a necessary factor for efficient LNP transfection. Further, they identify a relative high content of apolipoproteins and lipids associated with lipoproteins in the LNP samples isolated from LP and OP. These findings imply either that the LNPs are associated with lipoproteins, fuse with the lipoproteins, the co-isolation of individual lipoproteins.

Moreover, it is surprising that the LNP size increases from ~80 nm to ~150 nm and ~250 nm when exposed to 1% LP and 1% OP, respectively, based on dynamic light scattering (DLS) data. Such huge increase in LNP size is not expected for a protein corona layer on LNPs and only possible if LNPs form aggregates, the LNPs associate with larger particles (e.g. lipoproteins and EVs), the LNPs fuse together or with endogenous NPs, or the sample is contaminated with larger sized particles (e.g. lipoproteins and EVs). Cryo-TEM micrographs of the naked LNPs (LNPs in buffer that has not been exposed to plasma) and LNPs exposed to OP (Fig. 1c and d, respectively)^1^ support an increase in particle size in OP. The size increase is however likely not due to a thick protein layer, nor are LNP-based aggregates observed. The cryo-TEM micrograph of LNPs isolated from OP shows the appearance of unilamellar vesicle-like structures that resemble various extracellular vesicle morphologies. EVs have previously been ascribed a unilamellar morphology, where some may present as multiple overlapping or nested appearances and others as elongated or irregularly shaped^9^, features very similar to the structures shown in Fig. 1d^1^. This further supports the hypothesis that endogenous NPs may also be present in pull-down fractions. It should be mentioned that HDL and LDL are likely to small to be identified in the cryo-TEM micrographs.

Liu et al. suggest that the remodulation of the original dense and uniform LNPs into unilamellar vesicular-like structures is “likely resulting from incorporating biomolecules with detergent-like properties, such as apolipoproteins”. It is, however, known that lipid-free apolipoproteins can solubilize liposomes by transforming the lipids into nascent HDL-like structures^10^. Taken together, it is surprising that only 1% OP has such a great impact on the LNP structure. It is also surprising that these huge proposed LNP structural remodulations and size expansion do not give rise to a change in the FRET signal (Fig. 1e)^1^, an indirect measure of the LNP integrity, when compared to the naked LNPs in buffer. The FRET assay is based on a lipid-FRET donor and an mRNA-FRET acceptor FRET-pair. Hence, the FRET data combined with the DLS results and cryo-TEM observations could indicate that the LNPs exposed to OP are intact and that the presence of larger sized (endogenous) NPs give rise to the measured increase in the apparent LNP size.

My last concern relates to the particle concentrations that Liu et al. report (Fig. 1f)^1^ and the particle concentrations used in the in vitro and in vivo experiments. Liu et al. present particle concentrations of the HDL/IDL/LDL, VLDL/remnant chylomicrons (CMs), and CMs populations based on NTA measurements of the LP and OP plasma samples. First, it is important to note that NTA does likely not possess the sensitivity required to consistently detect lipid-based NPs below approximately 70 nm in size^11^. Some NTA equipment claims the ability to detect particles as small as 10 nm. However, it is essential to recognize that the detectable 10 nm NPs are likely made of metal, such as gold NPs, which scatter light significantly more than lipid-based NPs like LNPs. Second, HDL, LDL, and CM concentrations are ~10^16^, ~10^15^, and 1–2 × 10^13^ per ml human plasma, respectively^2^. Further, rats and many other species carry most of their plasma cholesterol in HDL, whereas cholesterol in humans has a preference for LDL^12^. Taken together, the relative particle concentrations of the different lipoprotein populations in rat plasma defined by Liu et al. would be expected to be the exact opposite to what is reported (see inserted table in Fig. 1f)^1^. Therefore, uncertainties are related to the actual HDL concentrations used in the biological studies, when most of the HDL will likely go undetected in NTA measurements, and to what extent a potential co-isolation of HDL or other types of endogenous NPs has on the biological studies presented by Liu et al. On this note, Liu et al. show data that support that the transfection potency of LNPs is highly dependent on plasma components in a non-linear fashion. Further, it has been reported that approximately half of total apoE is found on HDL^13^.

Taken together, the bulk data presented by Liu et al., including a negative control that is slightly positive, proteomic and lipodomic studies that identify lipoprotein components in the LNP isolate, and the strange non-LNP like structures and sizes observed with cryo-TEM, call for additional verification to support that their pull-down method does not pull down lipoproteins and EVs from plasma samples, in addition to the LNPs. Keep in mind that the isolation method relies on capturing PEG-lipids that are highly labile and which likely associate well with the lipid-surface on both LNPs, lipoproteins, and EVs.

To ensure that the isolation method being used does not co-isolate individual lipoproteins and EVs, I suggest a few control experiments: (i) Add PEG-lipids (like in case of the dot blot studies) to LP and OP, apply the pull-down LNP isolation method, and characterize the protein content, including performing apolipoprotein and EV maker (CD9, CD63, and CD81) immunoblotting analysis. This experiment serves as a control that could confirm whether the pull-down isolation method does not capture endogenous plasma NPs. (ii) Compare the protein content and composition of the LNPs captured after 20 and 30 min. incubation with the magnetic beads coated with PEG-antibodies in OP to clearly show that the decrease in LNP-capturing when going from 20 to 30 min. bead capturing incubation is not at the expense of an increase in the capturing of EVs and lipoproteins. (iii) I also suggest to employ A4F on the LNP isolates followed by proteomic or apolipoprotein immunoblotting analysis of the non-LNP A4F fractions to verify whether lipoprotein content is associated with or co-isolated with LNPs. Recently, Wu et al.^14^. nicely showed that HDL, LDL, and EVs in plasma can be well-separated by using A4F. Alternative, HDL and LDL could be separated from LNPs and larger sized endogenous NPs if present in the LNP isolate by using size-exclusion chromatography and analyzed.
